# Relationship between Personality Traits and Brain Reward Responses when Playing on a Team

**DOI:** 10.1371/journal.pone.0087277

**Published:** 2014-01-27

**Authors:** Carmen Morawetz, Evgeniya Kirilina, Juergen Baudewig, Hauke R. Heekeren

**Affiliations:** 1 Department of Education and Psychology, Freie Universität Berlin, Berlin, Germany; 2 Dahlem Institute for Neuroimaging of Emotion, Freie Universität Berlin, Berlin, Germany; 3 Biomedical Imaging, Department of Radiology, Christian-Albrecht University Kiel, Kiel, Germany; Chiba University Graduate School of Medicine, Japan

## Abstract

Cooperation is an integral part of human social life and we often build teams to achieve certain goals. However, very little is currently understood about emotions with regard to cooperation. Here, we investigated the impact of social context (playing alone versus playing on a team) on emotions while winning or losing a game. We hypothesized that activity in the reward network is modulated by the social context and that personality characteristics might impact team play. We conducted an event-related functional magnetic resonance imaging experiment that involved a simple game of dice. In the team condition, the participant played with a partner against another two-person team. In the single-player condition, the participant played alone against another player. Our results revealed that reward processing in the right amygdala was modulated by the social context. The main effect of outcome (gains versus losses) was associated with increased responses in the reward network. We also found that differences in the reward-related neural response due to social context were associated with specific personality traits. When playing on a team, increased activity in the amygdala during winning was a unique function of openness, while decreased activity in the ventromedial prefrontal cortex and ventral striatum during losing was associated with extraversion and conscientiousness, respectively. In conclusion, we provide evidence that working on a team influences the affective value of a negative outcome by attenuating the negative response associated with it in the amygdala. Our results also show that brain reward responses in a social context are affected by personality traits related to teamwork.

## Introduction

Cooperation is an integral part of human social life. Much of the work we do is accomplished through cooperation with others, and we often build teams to achieve certain goals. Humans cooperate in many ways and settings and to degrees that are unequalled among animals. Cooperation in the animal world challenges classical evolutionary theory by demonstrating that cooperation has evolutionary benefits through increasing survival fitness [1]–[5]. Cooperation broadly includes all forms of mutually beneficial joint action by two or more individuals [6] and arises from a variety of individual motivations, motives and dispositions [7]. A large body of research in cognitive neuroscience has investigated the neural underpinnings of social decision making through economic games [8], [9] that focus on cooperative mechanisms (e.g., [10]). We still know very little, however, about the relationship of emotions with cooperation and how cooperation affects emotional experiences. For example, we do not know if a successful team player feels more positive than a successful solo player. We also still know very little about the neural systems underlying cooperation and teamwork. Therefore the aim of the present study was to investigate how reward processing is modulated by cooperative behavior in terms of teamwork, how sharing gains and losses in a team context modulates affective responses and how certain personality characteristics and brain responses in reward-sensitive regions relate to each other in regard to cooperative behavior.

No fMRI study has yet investigated how cooperation is experienced in a team context and only a few behavioral and functional magnetic resonance imaging (fMRI) studies have investigated the neural underpinnings of cooperation and the influence of different social settings on reward processing. Voluntary cooperation is associated with self-reported pleasure and satisfaction [11] and is tightly linked to reward-related neural activity in the medial orbitofrontal cortex (OFC) [12]. Other researchers, using different versions of the trust game and the prisoner’s dilemma, have observed enhanced activity in the ventral striatum, rostral anterior cingulate cortex (ACC), medial OFC, ventral medial prefrontal cortex (vmPFC) and anterior insula during reciprocity [13]–[15], cooperation [16] and deciding to “share” or “keep” trust [17].

Few studies so far addressed the influence of social settings on reward processing. One example of how emotions are linked to social settings is provided by the observation that the relative weight of gains and losses differs according to the social setting [18]. Specifically, the experience and anticipation of losses loom larger than gains for private outcomes, whereas gains loom larger than losses in the social domain. A functional magnetic resonance imaging (fMRI) study also showed that activity in the ventral striatum decreases in response to social loss compared to private loss but increases during social gain compared to private gain [19]. Sharing a positive outcome with a close friend is associated with enhanced subjective feelings of excitement and enhanced striatal activity compared to sharing the outcome with an unknown confederate or a computer [20]. Competing against a close friend (compared to an unknown confederate or playing alone) leads to enhanced responses in the corticostriatal reward system, indicating that the medial PFC plays a key role in differentiating the outcome value in regard to the competitor, whereas the ventral striatum processes the outcome value in a more coarse sense [21].

The relationship between personality traits, teamwork and team effectiveness is another important aspect of cooperation. One of the most prominent concepts in personality research is the theory of the five-factor model of personality (Big Five), which assumes that personality can be described along five dimensions: Neuroticism, Extraversion, Openness to Experience, Agreeableness and Conscientiousness [22]–[24]. The personality traits of emotional stability, extraversion, openness, agreeableness and conscientiousness have all been broadly related to team effectiveness [25]–[29]. The influential role of personality characteristics that are related to cooperation has been supported by demonstrating a stronger link between cooperative behavior and personality traits, such as conscientiousness, extraversion and agreeableness, than between cooperative behavior and task performance [30]. Furthermore, there is increasing interest in neuroscience, particularly in neuroeconomics [31], in the study of individual differences in personality [32]–[34].

The primary goals of the present study were to investigate how the joint experience of a gain or loss influences an individual’s affective response and to examine the impact of individual differences on the sensitivity to a reward in relation to the social context. Thus, we designed a simple game of dice in which we manipulated the social context (playing alone or playing on a team of two). We used an implicit task in which the participants had to judge the visual pleasantness of a pictograph to measure affective reactions. We hypothesized that the blood oxygen level dependent (BOLD) signal in key structures of the reward system, including the ventral striatum, the amygdala, vmPFC, OFC and ACC [35]–[38], would be enhanced during winning compared to losing and when playing on a team compared to playing alone. We also put the hypothesis forward that an implicit measure of affect would indicate that participants feel more positive after a gain compared to a loss; and that the social context affects participants in such a way that they would feel more positive in the team compared to the single player condition. Finally, we propose that personality characteristics, such as extraversion, conscientiousness, openness and agreeableness, are reflected in the responsiveness of reward-sensitive regions and positively correlate with responses in reward-related regions in the team condition [39].

## Methods

### Ethics Statement

This study was approved by the local ethics committee at the Freie Universitaet Berlin, Germany. The study was carried out in accordance to the Declaration of Helsinki. Informed written consent was obtained from each subject before the study.

### Participants

A total of 39 right-handed, healthy volunteers (22 males, mean age = 24±4.06 years) participated in the study. Eleven of the subjects took part in a behavioral pilot (6 females, mean age = 21±2.11 years) and 28 in the fMRI experiment (11 females, mean age = 25±4.24). The design of the tasks performed in the behavioral and the fMRI experiments was identical. Handedness was assessed with the Edinburgh-Handedness Inventory [40], and eligibility was assessed with a general health questionnaire and an fMRI safety screening form. All participants had normal or corrected-to-normal vision and had no history of psychiatric or neurological disorder.

### Task Design

The experiment consisted of a simple game of dice. The objective of the game was to roll a number that was as close as possible to a predefined target number with either one die or two dice. The participants played this game either alone against another player (single-player condition) or with a partner against another two-person team (team condition). The study therefore involved four experimental conditions: gain and loss in the single-player condition and gain and loss in the team condition.

**Figure 1:**
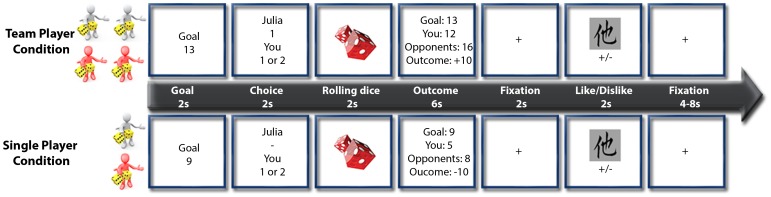
Experimental Design. Display of the team condition (first row) and single-player condition (second row). The goal of the game was to come as close to a predefined number with either one die or two dice. In the team condition, the participant played with a partner against another team consisting of two players. In the single-player condition, the participant played alone against another player. An event-related design was used. First the target number was presented for 2s. Then the participant had to choose either one die or two dice within 2s. In the team condition the participant was told how many dice the partner had picked. After the choice period, a short animation of rolling dice was presented for 2s. Finally, the outcome was revealed to the participant for 6s providing information about the goal of the trial, the result of both teams and most importantly the monetary gain or loss. This was followed by a fixation cross in the middle of the screen for 2s. In order to assess affect reactions, we implemented an implicit measure: A Chinese character was presented for 500ms and masked with a scrambled picture for 1500ms. Participants judged the visual pleasantness of each Chinese pictograph (like/dislike). Each trial was completed by a jittered fixation period of 4 to 8s displaying a fixation cross in the middle of the screen.

A mixed block/event-related design was employed (Figure 1) in which each block consisted of the experimental condition of either playing alone or as a team. A short instruction screen was presented for 2 s before each block that informed the participant of whether the following ten trials would be played in a single-player or team condition. Each trial began with a 2 s display showing the number (goal) that should be matched. The participant then had 2 s to make a choice to play with one die or two dice. In the team condition, the participant was told whether his/her team member picked one die or two dice. In the team condition, a maximum of four dice could be used to reach the goal (2 dice per player) while in the single-player condition a maximum of 2 dice could be used to play. Importantly, we programmed the experiment such that the teammate always behaved in a “cooperative” manner. This means, we “simulated” cooperative behavior in such a way that e.g. if the goal was lower than 13, only one die was picked, while e.g. if the goal was higher than 18, always two dice were picked by the teammate. The participant subsequently made his/her own choice based on this information. In the single-player condition, the participant did not have to consider the choice of his/her team member and could directly select the number of dice. An animation of rolling dice was presented for 2 s after the participant had made his/her choice. The outcome was then revealed, which consisted of information about the actual goal, the number achieved by the opposing team, the number achieved by the participant or the participants’ team, respectively, and the monetary gain or loss involved. This was followed by a fixation cross that was presented for 2 s in the middle of the screen. One of 160 different Chinese pictographs was next presented for 500 ms as an implicit measure of the participant’s affective reaction [41], and a mask consisting of a scrambled picture was subsequently presented for 1500 ms. The subjects had to judge the visual pleasantness of the Chinese pictograph by pressing a button that indicated whether they liked or disliked it and were instructed to respond quickly. Each trial ended with a jittered fixation period of 4 to 8 s with a fixation cross in the middle of the screen.

Each of the four runs consisted of four blocks. Each block included 10 trials per condition (winning and losing in the single-player condition and winning and losing in the team condition), for a total of 40 trials per run. Each run lasted approximately 13 min, and the blocks were presented in randomized order. Each block consisted of five winning and five losing trials, which were randomly distributed across the blocks. Participants could win or lose 5, 10 or 15 Euros. A maximum of 20 Euros was paid if the number was matched precisely. Neither participant nor team won or lost if there was a tie. The amount of the outcome of the trial was independent from the distance to the target number e.g. being closer to the target number did not imply a higher gain.

### Procedure

Participants were told that they would play an online game and that other players were located in separate rooms, when in fact, there were no other real players. The participants had to select a “team member” before they began the game. Photos of five women or men were presented, each with a fictitious name and age. The photos were taken from among the Karolinska Directed Emotional Faces (KDEF) displaying happy facial expressions [42]. The participants were asked to rate the other players regarding sympathy and how much they would like to play with them using a scale that ranged from –5 to +5. The photo of the person with the highest mean rating was selected as the participants’ team member based on the mean ratings recorded for the two questions. The name of the selected team member was used in the actual fMRI experiment and was displayed in each trial to increase the feelings of team membership. Female teammates were used for female participants and male teammates for male participants to minimize cross-gender effects that could influence social interaction. Participants were told that the teammate selection procedure based on the photos was being employed to minimize the interaction between the players.

The participants then had to choose between two playing cards to determine the sequence of players. They were told that if they chose a “1”, they would play first, and if they chose a “2”, they would play second. However, the cards were marked to ensure that every participant would always be the second player.

Before playing the game, each participant completed 3 practice trials to ensure that they understood the procedure. After each session, the participants were asked to pick a number between one and 160 to calculate their gain. The selected trial was randomly assigned to one of the winning trials from the complete fMRI session on which a participant’s payment was based. Each participant received 10 Euros for participating in the experiment and another 5 to 20 Euros depending on her/his choices. Thus, participants never really lost any money.

### Questionnaires

We administered the NEO-Five Factor Inventory (NEO-FFI) [43] to assess personality characteristics after the fMRI experiment. Participants also filled out a general questionnaire about the experiment in which they were asked to rate how much happiness and disappointment they had felt in response to gains and losses in the single-player condition and in the team condition on a Likert scale of 1 (not at all) to 5 (very) as well as some general questions about the experiment. Those general questions included: (1) “How important was the decision about the number of dice of your teammate to your own decision?” (Likert scale 1 to 5, 1: total rejection, 5: total agreement), (2) “I am convinced that I can achieve more in a game through cooperation.” (Likert scale 1 to 5, 1: total rejection, 5: total agreement), (3) “I perceive playing in a group predominantly as negative.” (Likert scale 1 to 5, 1: total rejection, 5: total agreement), (4) “How do you appraise the playing attitude of your teammate?” (open question).

### Behavioral Measures

Reaction times were measured when the participants had to choose between one die and two dice to allow us to verify that they believed that they were playing on a team. Reaction times should be longer if participants are playing on a team compared to when they are playing alone because they have to consider their partner’s response before making their own choice.

An implicit measure based on the affect misattribution procedure [41] was used to assess the participants’ affective reactions after the outcomes had been revealed to them. In order to measure unconscious representations that are inaccessible to introspection and to exclude the impact of social desirability we used an implicit affect measure [44], [45]. The affect misattribution procedure consisted of an affect-laden prime, followed by an ambiguous target. The influences of primes on target evaluations were used to assess participants’ attitudes toward prime objects. In this study, the outcome (gain or loss) was used as the prime, and Chinese pictographs were used as the targets to be judged based on their pleasantness. We expected that the participants would more often judge a pictograph to be pleasant after a positive outcome than they would after a negative outcome. The variable of primary interest was therefore the proportion of pictographs that the participants judged to be pleasant in each prime condition.

### Imaging Data Acquisition and Analyses

Whole-brain functional and anatomical images were acquired using a 3.0 T Magnetom TrioTim MRI scanner (Siemens, Erlangen, Germany) and a 12-channel head coil. A high-resolution 3D T1-weighted dataset was recorded for each participant (176 sagittal sections, 1×1×1 mm^3^; 256×256 data acquisition matrix). Functional images were acquired using a T2*-weighted, gradient-echo echo planar imaging (EPI) pulse sequence recording 37 sections oriented roughly parallel to the anterior commissure-posterior commissure line at an in-plane resolution of 3×3×3 mm^3^ (interslice gap = 0; TE = 30 ms; TR = 2 s; FA = 70°; FoV = 192×192 mm^2^; 64×64 data acquisition matrix). A total of 457 whole-brain volumes were recorded for each experimental run.

The obtained data were analyzed within the framework of a random effects general linear model (GLM) using BrainVoyager QX 2.6.0 (Brain Innovation, Maastricht, The Netherlands). The preprocessing of the fMRI data included 3D-motion correction, temporal high-pass filtering (3 cycles/run), linear trend removal, slice scan time correction, spatial smoothing (Gaussian smoothing kernel, 8 mm full width half maximum) and transformation into the space of Talairach and Tournoux [46]. Separate regressors in the GLM were specified for the goal, choice, dice rolling, outcome (divided into gains or losses in the single-player condition and gains or losses in the team condition) and affective responses. The length of the outcome phase was set to one second. One-sample t-tests were computed for different contrasts to assess random effects across participants. Corrections for multiple comparisons were performed at the cluster level through a Monte Carlo simulation [47], [48]. The uncorrected cluster threshold was set at p = 0.05. On the basis of the number of activated voxels and the estimated smoothness of the map (1.516), Monte Carlo simulations (1000 iterations) were performed to determine the minimum cluster size required to yield a maximum error rate of p<0.05 at the cluster level. The analysis was limited to *a priori* regions of interest (ROIs) using a mask defined through a topic-based search for a “reward” (topic 16) using neurosynth (http://neurosynth.org). This procedure revealed a meta-analysis map consisting of data from 100 studies (threshold of q(FDR) = 0.05) including the ventral striatum, amygdala, PCC and vmPFC. This map was used as a whole-brain mask for focused, hypothesis-driven analyses and was applied to the random effects GLM. We used the main effect of outcome to determine distinct clusters of activity within the mask, which were then used to define functional ROIs. ROI analyses were performed on the vmPFC (x = 2, y = 38, z = 10, size = 6,008 voxels), the ventral striatum (x = –1, y = 8, z = 5, size = 5,239 voxels), the amygdala (x = –25, y = –9, z = –12, size = 947 voxels; x = 20, y = –2, z = –6, size = 1,065 voxels) and the PCC (x = –1, y = –29, z = 33, size = 2,338 voxels).

*Post hoc* analyses were conducted through paired t-tests (two-tailed), repeated measures ANOVA, correlation analyses (Pearson’s r) (controlled for multiple comparisons using Bonferroni correction) and stepwise regression analysis using SPSS (Version 20). In the analyses of the behavioral data, the data from the behavioral pilot and those collected during the fMRI experiment were combined to increase statistical power (n = 38).

## Results

### Personality Results

The mean ratings of affect (happiness about a win and disappointment about a loss) revealed no impact of social context on the ratings. Generally, participants were not more happy after a win in the team compared to the single player condition (t(26) = 0, p = 1) and not less disappointed after a loss in the team compared to the single player condition (t(26) =  –0.18, p = 0.85). Importantly, however, participants indicated that the decision of the teammate was important to their own decision (question 1) (M = 4.15, SD = 0.67). Participants also strongly agreed that cooperating in a game is of great relevance to them (question 2) (M = 4.15, SD = 0.92) and playing in a group is not perceived negatively (question 3) (M = 1.73, SD = 0.66). In the open question about the playing attitude of their teammate (question 4), participants described their teammate’s behavior as e.g. “reasoned”, “conservative”, “good”, “normal”, “cooperative”, “helpful”, “careful”, and “clever”. Only one participant presumed that he was playing with a computer.

We correlated the different personality traits of the NEO-FFI with the items obtained from the general questionnaire about the experiment (questions 1–4) and the behavioral results of the implicit affect measure (proportion “pleasant” responses). Neuroticism correlated negatively with question 1 (r = –.41, p = 0.03), extraversion correlated positively with question 1 (r = .39, p = 0.04) as well as question 2 (r = .54, p = 0.004) and negatively with question 3 (r = –.64, p<0.001). Openness to experience correlated positively with question 2 (r = .49, p = 0.01) and negatively with question 3 (–.41, p = 0.03). No significant correlations were observed between the personality traits and the implicit affect measure after winning and losing.

### Behavioral Results

**Figure 2:**
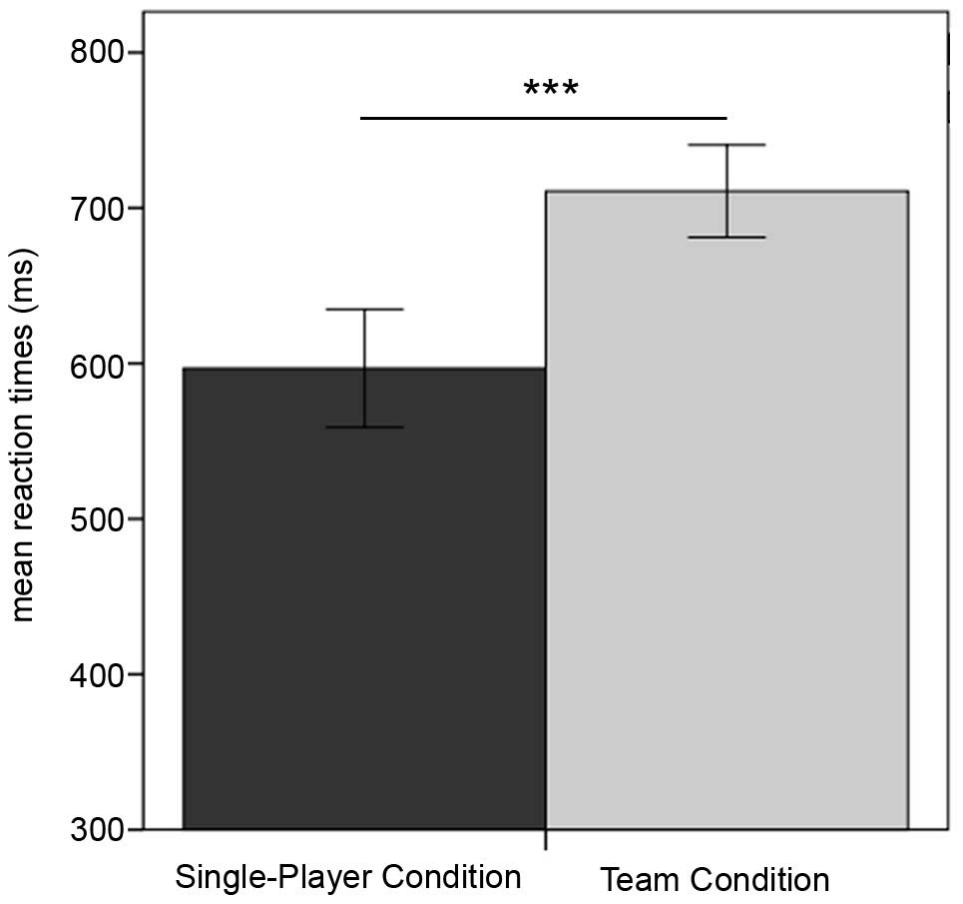
Reaction Times. Mean reaction times for choice period for single-player and team condition. Error bars represent one standard error. ***Indicates significant difference between conditions at p<0.001.

The mean reaction times associated with choosing the number of dice were computed separately for each participant and for the single-player condition and the team condition. The participants took significantly longer to make their choice in the team condition (t(37) =  –7.43, p<0.001) (Figure 2).

**Figure 3:**
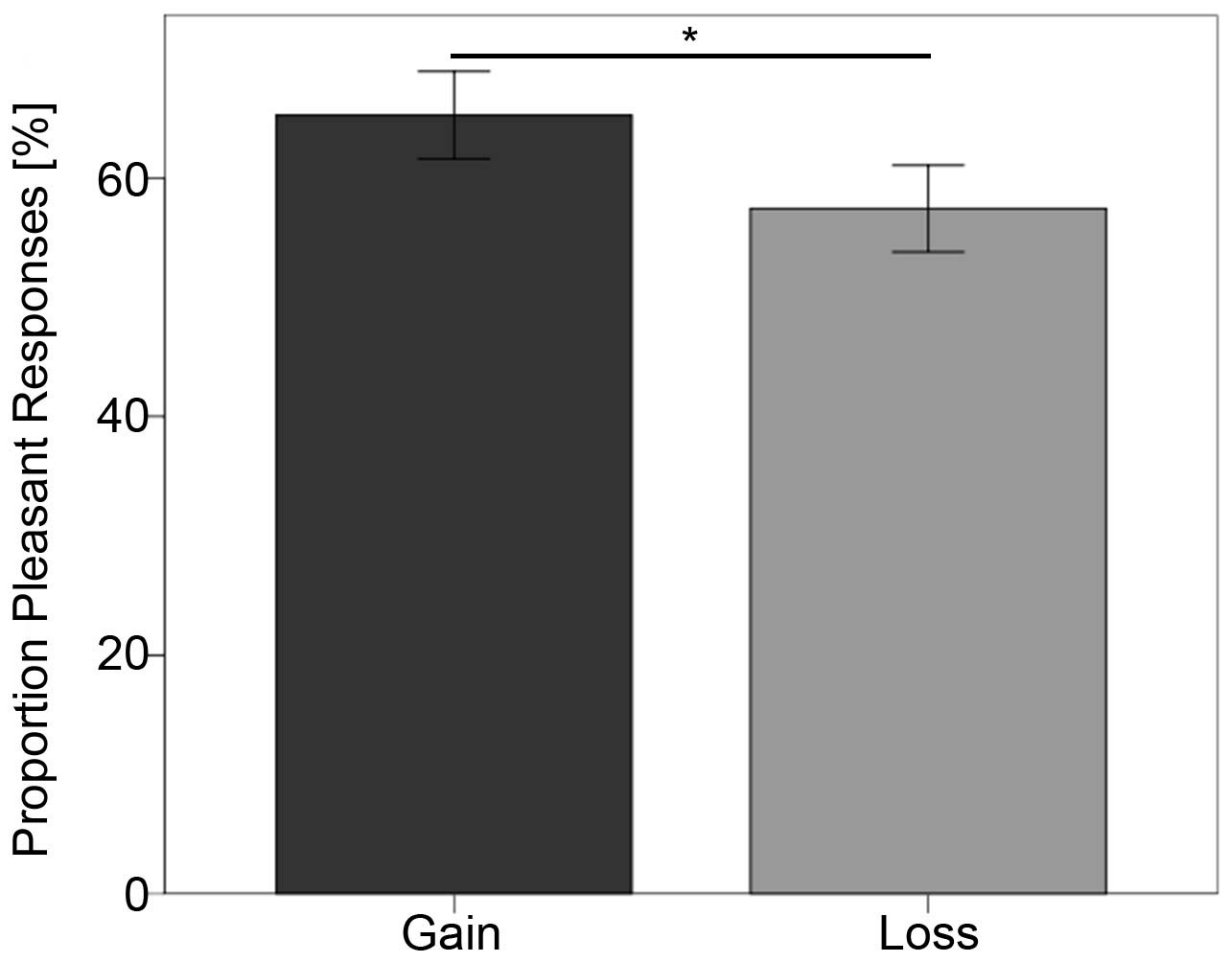
Implicit Affect Measure. Proportion of “pleasant” responses as a function of outcome. Error bars represent one standard error. *Indicates significant difference between conditions at p<0.05.

Repeated measures ANOVA [2 (outcome: gain/loss) x 2 (setting: single-player condition/team condition) x 2 (experiment: behavioral pilot/fMRI experiment)] of the results of the implicit measure revealed no significant main effect of experiment (F(1,10) = 0.35, p = 0.35) and no interaction effects with one of the other factors. Therefore, the behavioral data of the behavioral pilot and the fMRI experiment were combined. Repeated measures ANOVA [2 (outcome: gain/loss) x 2 (setting: single-player condition/team condition)] of factors that influenced the implicit measure revealed a clear main effect of outcome (F(1,38) = 5.62, p = 0.02), no main effect of setting (F(1,38) = 0.25, p = 0.61) and no interaction effect of outcome and setting (F(1,38) = 0.09, p = 0.76). The participants were more likely to judge the Chinese pictographs as pleasant and liked them more following a win and were less likely to judge them as pleasant and disliked them more following a loss (t(38) = 2.37, p = 0.02) (see Figure 3).

### fMRI Results

**Figure 4:**
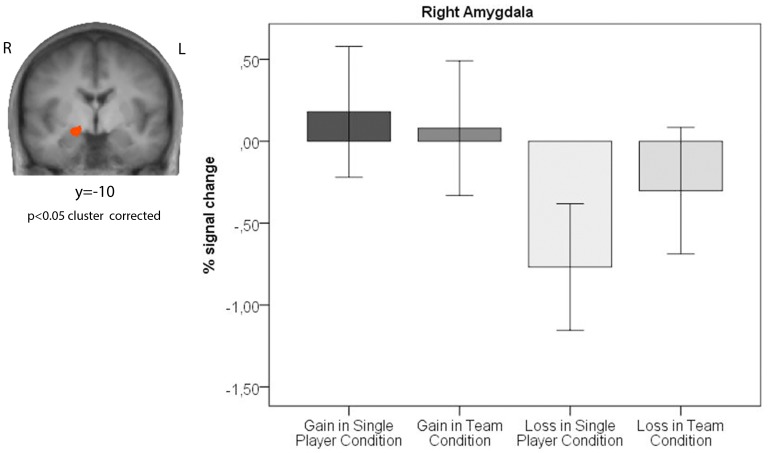
Amygdala activity encoding relative valence and context of outcomes during the outcome evaluation period. The coronal slice shows the interaction effect between outcome (gain/loss) and social setting (single-player condition/team condition) in the right amygdala. The bar graphs indicate the percent signal change (±SE) for the right amygdala (x = 20, y = –1, z = –7; size = 1065 voxels).

A repeated-measures ANOVA [2 (outcome: gain/loss) x 2 (setting: single-player condition/team condition)] revealed an interaction effect (F(1,27) = 2.24, p = 0.01) and a main effect of outcome (F(1,27) = 17.56, p<0.001) indicating enhanced activity in response to gains compared to losses in the right amygdala (Figure 4), but no main effect of setting (F(1,27) = 3.43, p = 0.07). Losing in the single-player condition was associated with lower activity in the right amygdala than losing in the team condition (t(27) =  –3.59, p = 0.001). Winning in the single-player condition was related to increased activity in the right amygdala compared to losing in the same condition (t(27) = 5.02, p>0.001). The same result was observed for the team condition (t(27) = 1.97, p = 0.05).

**Figure 5:**
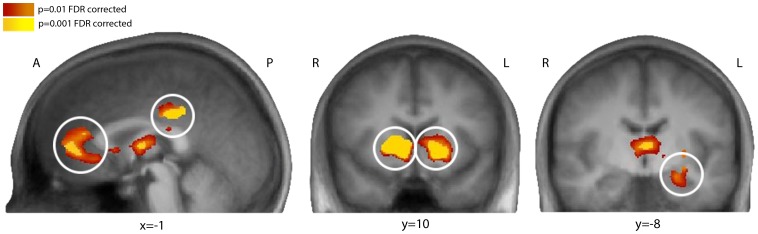
Activity in reward-related regions during the outcome period. The main effect of outcome was associated with increased activity in vmPFC (x = 1, y = 38, z = 10; size = 6008 voxels, PCC (x = –1, y = –29, z = 33; size = 2338 voxels) displayed in the sagittal view on the left. The coronal slice in the middle shows increased activity in bilateral ventral striatum in response to outcome (x = –1, y = 8, z = 5; size = 5239 voxels). The coronal slice on the right shows enhanced signal change in the left amygdala during the outcome phase (x = –25, y = –9, z = –13; size = 947voxels). A = anterior. P = posterior. R = right. L = left.

**Figure 6:**
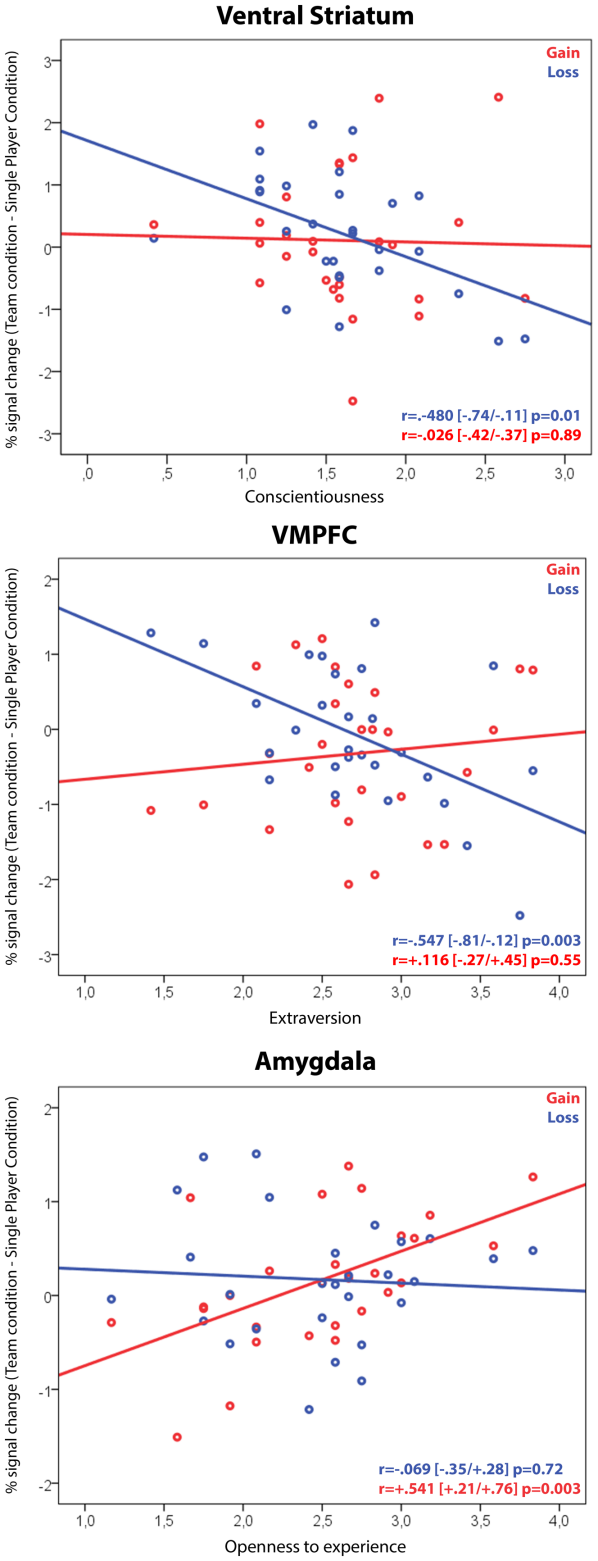
Correlations between differential activity during the outcome phase and personality characteristics. Each data point represents a measurement from one participant. The solid black lines indicate the linear regression for each panel. Correlation coefficients and statistical significance are denoted in the lower right corner of each panel for gain and loss separately. The numbers in brackets denote the bootstrapped 95% confidence intervals for each correlation coefficient.

As expected, the main effect of the outcome was associated with increased activity in reward-related regions, such as the vmPFC, bilateral ventral striatum, left amygdala and PCC (Figure 5, Table 1). To further explore the relationship between personality characteristics and the BOLD signal change observed in relation to social conditions, we correlated various personality traits (extraversion, openness to experience, neuroticism, conscientiousness and agreeableness) with the BOLD signal recorded during the outcome phase in the ROIs (note that ROIs were identified independent of personality traits) (Figure 6). We specifically investigated whether individual differences in these personality traits were correlated with the magnitude of the reward response. We defined the difference in the magnitude of the reward response as the difference in activation as a function of the outcome in each ROI during the team condition and the single-player condition. A difference in activation in the vmPFC (activation in the team condition minus activation in the single-player condition) was negatively correlated with extraversion during a loss situation compared to a win situation (loss: r = –.55, p = 0.003; gain: r = .12, p = 0.55; Fishers’ z = –2.52, p<0.05). Conscientiousness was also negatively correlated with the difference in activation in the ventral striatum during a loss situation (loss: r = –.48, p = 0.01; gain: –.03, p = 0.89; Fishers’ z = –1.77, p<0.05). Openness to experience was positively correlated with a differential BOLD signal in the left amygdala during a win situation (gain: r = .54, p = 0.003; loss: r = –.07, p = 0.72; Fishers’ z = –2.34, p<0.05).

**Table 1:** Cluster Peak Table for main effect outcome.

Region	L/R	F-value	Coordinates		
			x	y	z
Ventromedial PFC	R	28.03	2	40	6
Posterior Cingulate Gyrus	R	42.37	2	–26	29
Nucleus Caudatus	R	87.43	11	10	3
	L	49.81	–13	10	1
Amygdala	L	23.55	–22	–8	–12

p<0.001 FDR corrected.

We then performed a multiple regression analysis to predict the relationships between personality characteristics and neural activity. We conducted a multiple stepwise regression to examine the effects on the BOLD signal independently in each ROI in association with the different social settings and outcomes while statistically controlling for the other reward-related ROIs. Each personality trait was entered as a dependent variable, and the differences in BOLD signal changes (activation in the team condition minus activation in the single-player condition) observed in each ROI during winning and losing represented the independent variables. We applied the stepwise method and found a significant model for extraversion (F(1,27) = 11.12, p = 0.003, adjusted R^2^ = .273). The only significant predictor variable was the BOLD signal change in the vmPFC during a loss condition (β = –.547, p = 0.003). Tests for multicollinearity indicated the presence of a very low level of multicollinearity (vmPFC-gain: VIF = 1.03, amygdala-gain: VIF = 1.0, amygdala-loss: VIF = 1.42, ventral striatum-gain: VIF = 1.0, ventral striatum-loss: VIF = 1.23, PCC-gain: VIF = 1.01, PCC-loss: VIF = 1.48). The multiple regression analysis also revealed a significant model for conscientiousness (F(1,27) = 7.79, p = 0.01, adjusted R^2^ = .201) that was uniquely predicted by the BOLD signal changes within the ventral striatum during losing (β = –.480, p = 0.01). Tests for multicollinearity indicated the presence of a very low level of multicollinearity (vmPFC-gain: VIF = 1.0, vmPFC-loss: VIF = 1.23, amygdala-gain: VIF = 1.0, amygdala-loss: VIF = 1.13, ventral striatum-gain: VIF = 1.0, PCC-gain: VIF = 1.01, PCC-loss: VIF = 1.61). The BOLD responses recorded in the left amygdala were significantly associated with openness to experience (F(1,27) = 10.47, p = 0.003, adjusted R^2^ = .265) during winning (β = .541, p = 0.003). Tests for multicollinearity indicated the presence of a very low level of multicollinearity (vmPFC-gain: VIF = 1.56, vmPFC-loss: VIF = 1.0, amygdala-loss: VIF = 1.0, ventral striatum-gain: VIF = 1.47, ventral striatum-loss: VIF = 1.0, PCC-gain: VIF = 1.37, PCC-loss: VIF = 1.03). Neuroticism (F(1,27) = 4.71, p = 0.04, adjusted R^2^ = .121) was solely predicted by signal changes in the left amygdala during the loss condition (β = .392, p = 0.04). Tests for multicollinearity indicated the presence of a very low level of multicollinearity (vmPFC-gain: VIF = 1.07, vmPFC-loss: VIF = 1.42, amygdala-gain: VIF = 1.0, ventral striatum-gain: VIF = 1.0, ventral striatum-loss: VIF = 1.13, PCC-gain: VIF = 1.02, PCC-loss: VIF = 1.32). There were no significant associations between agreeableness and the BOLD responses observed in any of the ROIs.

## Discussion

The present study used a dice game to investigate the effects of cooperation and the social context on reward responses. We compared how positive and negative outcomes are processed when a person plays as part of a team versus playing alone, respectively, and the impact of personality traits on teamwork. Our results suggest that receiving a negative outcome together with a teammate elicits a smaller decrease in BOLD signal in the right amygdala than when receiving it alone. The differential activity in several regions within the reward network between winning and losing on a team versus alone correlated with certain personality traits suggesting that personality characteristics may play an important role in the processing of reward in different social settings.

We found that reward processing in the right amygdala was modulated by the social context. The amygdala encoded both positive and negative outcomes modulated by the social context with increased activity observed in response to gains compared to losses and an amplified negative response to losses recorded in the single-player condition compared to the team condition. The amygdala has been assigned the important role within the reward network of encoding the affective significance and subjective relevance of a stimulus, and it processes positive and negative emotions similarly [37], [49], [50], leading to the conclusion that amygdala activity is linked to the contextual and goal-dependent value of a stimulus in a personal situation [51]. The present results confirm the hypothesis that the amygdala is involved in the emotional aspects of reward by showing that the amygdala differentially assigns value to negative outcomes depending on the social context. In contrast to the results of previous studies, we observed an effect of the social context on reward processing in the right amygdala but not in the ventral striatum [19]–[21]. This discrepancy may have been due to different task designs. Bault et al. [19] employed a lottery choice, and Fareri et al. [20], [21] applied a card-guessing task, with the participants observing a positive or negative outcome in different social settings under both of these methods. The participants in these studies did not cooperate with other players to achieve a goal and did not compete against another team. They simply compared their own outcome to the outcome of the other players passively, implying the involvement of social comparison and social ranking, which have previously been associated with activity in the ventral striatum [52], [53]. The present study focused more specifically on the emotional consequence of sharing a win or a loss in a team context, and emphasized the specific role of the amygdala in reward processing by adding a social and affective component to the outcome.

In line with previous findings, the main effect of the outcome revealed increased BOLD responses in the reward network comparing gains with losses [35], [54]. We were also interested in how specific personality traits relate to reward responses in different social settings. We found that the differential neural responses observed in reward-related regions when playing on a team compared to playing alone were associated with specific personality traits. Specifically, increased activity in the amygdala during winning on a team was a unique function of openness, whereas decreased activity in the vmPFC during losing on a team was predominantly associated with extraversion, and decreased activity in the ventral striatum during losing on a team was predominantly associated with conscientiousness.

Our findings revealed a relationship between extraversion and vmPFC activity that was modulated by the social context. Extraverts not only indicated a high level of interest in social cooperation (question 2) but also demonstrated high consideration of their teammates’ decision (question 1). This leads us to infer that for participants who are skilled in considering the perspective of others, e.g., being considerate of a teammate’s position and being highly interested in social interaction, a loss under team conditions looms larger than a loss under single-player conditions. Several studies have established that extraversion is connected to reward sensitivity and reward anticipation [34], [55]–[59] and have established the vmPFC as a key component in the reward circuitry. vmPFC activity has been linked to a wide range of valuation and choice signals [60] and to value-guided decision-making by monitoring and evaluating reward outcomes [61], including both social and monetary rewards [54]. Two previous studies have revealed modulation of vmPFC responses to positive and negative outcomes according to the social context [20], [21]. In addition to its role in reward processing, the vmPFC has been implicated in “theory-of-mind” abilities [62], [63] and in empathy [64], thus providing a link to the correlation observed in the present study between extraversion in the form of social perceptiveness and vmPFC activation.

The results of the present study yielded an exclusive negative correlation between conscientiousness and a decrease in striatal activity during a loss in a team context compared to a loss under single-player conditions. Many neuroimaging studies have implicated the ventral striatum in reward processing, specifically in encoding stimulus-reward value and reward prediction [35], [38], [61], [65]–[70]. The ventral striatum was observed to encode positive and negative outcomes in the present study, which is consistent with results of previous studies [19]–[21]. However, in contrast to previous results, we did not observe an effect of the social context on changes in BOLD signal in the ventral striatum [19], [20], which might be related to the different task designs. The association between conscientiousness and ventral striatum activity is supported by previous results that reported a link between individual differences in persistence and conscientiousness and specific brain areas, including the ventral striatum [71].

We also found a main effect of the outcome in the amygdala, highlighting the important role of this structure in reward processing, which is consistent with previous findings [35]. Openness to experience correlated positively with activity in the left amygdala during winning when playing on a team compared to when playing alone. Amygdala activity has mainly been related to neuroticism, anxiety and a negative affect [34], rather than to approach-related personality characteristics, such as extraversion and openness. However, personality measures associated with the behavioral approach system [72], which is closely related to openness to experience [22], [73], have been reported to be positively correlated with amygdala activity occurring when positive stimuli are presented [74]. A resting state study also found that regional activity of the amygdala correlated positively with openness [75].

Longer reaction times were observed in the team condition compared to the single-player condition, which indicated that the participants did, in fact, consider the choice of their teammate before making their own decision. This is further supported by the post-session ratings indicating that the decision of the teammate was important for the own decision. The playing attitude of the teammate was also described in positive terms suggesting that the experimental modulation of “cooperative behavior” was accepted by the participants. However, we cannot rule out the fact that longer reaction times in the team condition might be due to a heightened level of difficulty induced by the increased number of dice resulting in higher cognitive demands.

The post-session ratings of the participants of their experiences during the different task conditions revealed no significant difference in happiness about a win or disappointment about a loss in the team and single-player condition. This is in line with previous studies showing no effect of social context on excitement and disappointment ratings for sharing positive or negative outcomes in a card guessing task with either a confederate or a computer [20]. In the study of Fareri et al. [20] participants were only more excited to win when they were playing with a friend, suggesting that the affect associated with a reward depends upon the social role of the team member. Furthermore, we used a task where the cooperative condition provided no material benefit to participants. Participants’ probability of winning in a team was the same as in the single-player condition. Thus, cooperation is not more desirable or advantageous in the current game. This might explain why we did not observe any difference in the post-session ratings.

Importantly, we found associations between certain personality traits and the questions concerning the experiment and playing attitude. For participants scoring high on neuroticism, the decision made by their teammate was less important than for participants scoring low on neuroticism, i.e. emotionally stable participants. This indicates that participants who are more anxious, irritable, insecure, lacking self-esteem, and nervous [76] don’t value their teammates’ decision so much because they doubt that they will succeed and focus on avoiding failure [77]. In contrast to highly extraverted participants the teammate’s decision was of great importance. This is in line with the assumption that extraverts like to engage in activities with others, are very sociable and interested in social interaction as well as show high social perceptiveness [76], which is related to social understanding and empathy [39]. The finding that participants scoring high on extraversion and openness to experience, think that they can achieve more in a game when cooperating instead of playing alone and that playing in a group makes them feel positive underlines the positive relationship between those two personality traits, teamwork and cooperation [39].

The behavioral results obtained regarding the implicit affect measure confirmed our hypothesis that the participants were more likely to judge a pictograph as pleasant following a win and less likely to do so following a loss. This finding shows that the affective valence of the outcome influenced the participants’ evaluations of the pictographs and that even during the simple task employed here, winning was associated with a positive affect and losing with a negative affect. However, we did not observe any significant effects of social context and personality traits on the implicit affect measure. Participants indicated either if they liked or disliked a Chinese character, which represents a very coarse measure of affect that might not be suitable to capture the effect of social context. A more differentiated scale might have been better to determine the small effect of social context and the impact of personality traits on the implicit affect measure.

There are some aspects of the current study that could be addressed in future work. We did not include a nonsocial computer condition as control. However, it would be interesting to directly compare cooperation/teamwork in a social and nonsocial setting. Future work could also consider introducing another social condition by using a close friend as team member compared to an unknown confederate similar to a previous study [20]. We believe that this might influence the affective experience during the experiment. Another interesting issue concerns the sequence of playing e.g. that the participant would not always be the second player to pick a die but also the first one on some trials. A variation in the playing sequence could increase the feeling of social cooperation and make the game more “realistic”. Furthermore, feedback trials could be implemented in the game, in which participants could exchange their emotions with their team member in order to assess the emotional responses in a more direct way instead of using an implicit affect measure.

Finally, our findings constitute an important contribution to the understanding of cooperative behavior, how it is represented in the brain and modulated by the social context and certain personality characteristics. With regard to identity neuroeconomics, which aims to understand social motivations and their dependency on social identities and the social context [78], the present work extends previous studies employing economic games to investigate social decision making in a generic social context. We implemented a higher level of social context, known as the identity level [78], in which participants are divided into groups whose members care about their own and others’ actions. Our study, therefore, represents a first step toward incorporating meaningful relationships into experimental paradigms of social decision making [78] and offers the first insights into the implications of individual differences in personality for reward processing during cooperative behavior when playing on a team. Crucial findings in our study are the role of the right amygdala in the processing of negative outcomes which are received alone compared to a social team condition and the observation that activity during winning/losing alone/in a team in reward-sensitive regions including the ventral striatum, the VMPFC and amygdala correlated with conscientiousness, extraversion and openness to experience. These findings suggest that working on a team influences the affective value of a negative outcome by attenuating the negative response associated with it in the amygdala. Finally, it is important to note that brain reward responses in a social context are affected by personality traits important for teamwork.
